# Cardiac Involvement in Monkeypox Outbreak

**DOI:** 10.15190/d.2023.10

**Published:** 2023-09-18

**Authors:** Muhammad Romail Manan, Iqra Nawaz, Fatima Zafar, Hamna Manan, Yashfa Nawaz

**Affiliations:** ^1^Services Institute of Medical Sciences, Lahore, Pakistan; ^2^Quaid-e-Azam Medical College, Bahawalpur, Pakistan; ^3^Masood Hospital, Lahore, Pakistan; ^4^Capital Development Authority Hospital, Islamabad, Pakistan

**Keywords:** Monkeypox, Myocarditis, Pericarditis, Heart, Smallpox.

## Abstract

Unusual presentations and uncommon clinical manifestations of Monkeypox (Mpox) in the current outbreak highlight the need to focus on cardiac symptoms of the virus. Owing to limited discussion regarding cardiac involvement in recent cases of Mpox, we conducted a scoping review to determine the range of existing research and provide a descriptive overview of the current literature on these manifestations. This review was conducted using a previously developed six-stage methodological approach and keeping in view the Preferred Reporting Items for Systematic Reviews and Meta-analyses extension for Scoping Reviews (PRISMA-ScR). Records retrieved from PubMed, ScienceDirect and Google Scholar, using a two-step search strategy, were subjected to title and abstract screening, followed by full text screening of remaining articles against specified eligibility criteria. Relevant information was extracted and summarized. Our search yielded 707 records. Following title and abstract screening, 23 articles were retrieved for full text screening. Finally, a total of nine articles were included in this review (three case series and six case reports discussing a total of 13 patients). Myocarditis was identified as the most frequently reported cardiac manifestation of Mpox. Novel clinical presentations included pharyngitis, sore throat, proctalgia, and perianal irritation. Most patients reported chest pain as the primary symptom of cardiac system involvement. Elevated troponin was the most commonly reported investigation finding followed by an elevated C- Reactive Protein. There exists a lack of high-quality studies investigating cardiac system involvement in the current outbreak of Mpox. More information is needed regarding risk factors for cardiac complications, disease progression, and cardio tropism and immunological response to improve preventive/therapeutic strategies. We highlight the paucity of relevant data and call for further discussion to improve the understanding of cardiac manifestations of Mpox. This scoping review sheds light on the underexplored cardiac manifestations of Mpox and highlights the need for heightened awareness of cardiac symptoms in the current outbreak.

## SUMMARY

*1. Introduction*

*2. Identifying Relevant Studies*

*3. Study Selection Process*

*4. Data Charting*

*5. Collating, Summarizing, and Reporting the Results*

*6. Search Results and Characteristics of Included Studies*

*7. Patient Characteristics*

*8. History of Sexual Contact*

*9. Clinical Presentation*

*10. Cardiac Manifestations*

*11. Investigation Findings*

*12. Diagnosis*

*13. Management*

*14. Discussion*

- * 14.1 Cardiovascular events following vaccination*

- * 14.2 Cardiac Manifestations of Mpox*
  - *14.2.1 Pathogenesis of Mpox-induced Myocarditis*
  - *14.2.2 Pathogenesis of Mpox-induced Pericarditis*
- * 14.3 Investigations and Management*

- * 14.4 Limitations*

- * 14.5 Future Recommendations*

*15. Conclusion*

## 1. Introduction

As the world had begun to recover from the damage coronavirus disease 2019 (COVID-19) had caused, health systems around the globe were faced with another emerging infection the cases of which, although date back to the 1950s, became a public health emergency of global concern only recently. As of January 31, 2023, over eighty-five thousand people in 110 countries have been reported to be infected with Monkeypox (Mpox)^1^. Only seven of these countries, in the region of Central and West Africa, have had reports of Mpox in the past^2^. Over the past couple of months, as the spread of the disease intensified and became more widespread, more than ten thousand new cases have been documented and four new geographical locations have been impacted^2^. With the emergence of cases in non-endemic regions, the World Health Organization (WHO) declared it a global health emergency in July 2022, having issued an alert two months earlier in May^2^. Since then, reports of cases in new locations have uncovered clusters of novel clinical manifestations that provide important insight into the clinicopathological differences of the current cases from the previous ones.

Patients with Mpox infection typically report nonspecific prodromal symptoms such as fever, fatigue, malaise, and headache, however, in the current outbreak, presentation with classic lesions without a prodromal phase is not uncommon^2^. The cutaneous lesion classically presents as singular vesicles or a cluster of painful umblicated papules having centrifugal pattern of distribution with local lymphadenopathy and a myriad of systemic symptoms^2^. However, skin lesions with unusual distribution and characteristics have been documented over the genitalia, anus, and perianal area. Additionally, anorectal manifestations such as proctitis, tenesmus, diarrhea, and proctalgia have also been reported^2^. Similarly, pharyngitis, tonsillar lesions, and epiglottitis are some of the oropharyngeal presentations of the novel Mpox outbreak^2^. Other uncommon presentations include ocular lesions such as conjunctivitis^3^, as well as certain cardiovascular manifestations. As cardiac symptoms of Mpox remain underdiscussed, we performed a scoping review to assess the extent, range, and nature of available research and to provide a descriptive summary of the existing literature on these manifestations using a systematic approach.

This review was undertaken using the six-stage methodological approach developed by Arksey and O’Malley^4^, presented in their 2005 guidelines titled ‘Scoping studies: Towards a methodological framework’, which is also in compliance with the Preferred Reporting Items for Systematic Reviews and Meta-analyses extension for Scoping Reviews (PRISMA-ScR). With reports of unusual presentations and uncommon clinical manifestations of Mpox, a broad review question, in line with the recommendations for scoping reviews, was developed to guide the course of this review: What are the manifestations of cardiac involvement in patients diagnosed with Mpox in the current outbreak? This scoping review only included previously published scientific literature, without direct involvement of any patient population, therefore, ethical committee approval was not required.

## 2. Identifying Relevant Studies

In order to identify articles reporting cardiac manifestations in Mpox patients, a thorough literature search was performed on the following databases: PubMed, ScienceDirect, and Google Scholar using a two-step search strategy. First, an initial search of the information sources listed above was undertaken using key terms, modified accordingly for each database, separately and in combination. The search string employed to retrieve relevant data on PubMed is provided in Table 1.

**Table 1:** Search string for retrieving relevant records on PubMed

Concepts	Search String
Cardiac manifestations	(heart) OR (cardiac) OR (st segment) OR (heart failure) OR (cardiogenic shock) OR (myocarditis) OR (pericarditis) OR (acute coronary syndrome)
Monkeypox virus	(monkeypox) OR (monkeypox virus) OR (monkey pox) OR (mpox) OR (mpxv)

In the next step, citation lists of relevant studies were manually screened to develop a comprehensive list of literature eligible to proceed to the screening process.

## 3. Study Selection Process

**Figure 1:**
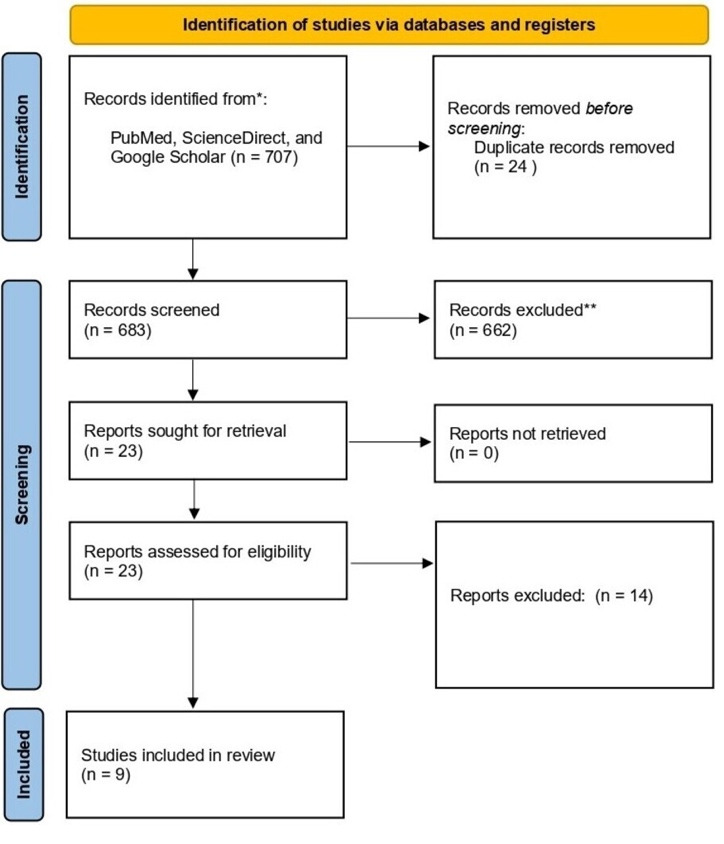
Preferred Reporting Items for Systematic Reviews and Meta- analyses (PRISMA) flowchart showing the article screening process

Eligibility criteria: Primary literature, published in the English language from January 2022 to January 2023, that reported cardiac manifestations in patients of Mpox, was considered eligible for inclusion without any restrictions regarding race, geographical location, gender, or setting. Letters, correspondence, opinions, perspectives, and book chapters were excluded. Studies evaluating cardiac complications of monkeypox vaccines were also not included. Before each phase, a pilot stage was conducted to ensure understanding of eligibility criteria between reviewers and the level of agreement was measured using the Cohen’s Kappa with a value of >0.8 required to initiate the complete screening process. Study screening process: Following the removal of duplicates, screening of extracted articles was carried out using a two-step process. During the first phase of screening, title/abstract of extracted studies were screened independently and in duplicate by two authors using Rayyan. In case of any disagreement during title/abstract screening, the article was included. Title/abstract screening was followed by full-text screening of the remaining articles. Any disagreement during the second phase of screening was resolved through mutual discussion and consensus. The Preferred Reporting Items for Systematic Reviews and Meta-analyses (PRISMA) flowchart developed to systematically present the article screening process has been shown in Figure 1.

## 4. Data Charting

A structured data extraction form was developed on Microsoft Excel and checked independently by two reviewers for clarity and relevance. Key data points from each article were extracted which included the Author name, article type, and country of affiliation of corresponding author. Data pertaining to the objectives of this scoping review included; age of the patient, sex of the patient, presenting complaint, characteristics of cutaneous lesions, sexual history, comorbidities, previous and concurrent sexually transmitted diseases (STDs), investigation findings, diagnosis, management, duration of hospital stay, and patient outcome.

## 5. Collating, Summarizing, and Reporting the results

The final version of the summary of findings extracted was revised to include feedback from all co-authors and results obtained are discussed in detail under the relevant heading.

## 6. Search Results and Characteristics of Included studies

Using the two-step search strategy, our search yielded a total of 707 records from the three databases. Following the removal of duplicates, a total of 683 articles were considered eligible for screening. Of these, 662 articles failing to meet the eligibility criteria were excluded, and 23 articles were retrieved for full-text screening. Finally, a total of nine articles were included in this review, which included three case series^5-7^, and six case reports^8-13^, discussing a total of 13 patients. Out of these articles, two each were published from Canada and the United States of America, while one article each was published from institutions based in France, Portugal, Italy, Spain, and Pakistan. Summary of all 13 cases has been presented in Table 2 and Table 3.

**Table 2:** Summary of demographic characteristics, presentation, and medical history of 13 patients discussed in our review

Cases	Age (years)	Sex	History of COVID-19	Presentation	Fever	Myalgia	Malaise	Head-ache	Lymph-adenopathy	History of recent sexual contact	PrEP Use	Co-morbidities	Previous STDs	Concurrent STDs
Case 1^8^	34	M	Negative	Fever, chills, and chest pain	Yes	Yes	NR	NR	B/L tender Inguinal	Heterosexual Also history of contact with potentially contaminated fomites	None	None	None	Chlamydia
Case 2^9^	31	M	Positive	Malaise, myalgias, and fever followed by eruption of multiple cutaneous lesions.	Yes	Yes	Yes	NR	Lt. inguinal	NR	Yes	None	None	None
Case 3^12^	51	M	NR	Chest Pain	Yes	-	Yes	NR	None	NR	None	None	None	None
Case 4^13^	>40	M	NR	Skin lesions, constitutional symptoms	Yes	Yes	NR	Yes	Rt. tender Sub-mandibular	MSM, engaged in oral sex, rimming, and condomless, insertive anal sex	None	None	None	Stable HIV
Case 5^6^	32	M	Negative	Chest pain and dyspnoea	NR	NR	Yes	Yes	Cervical Left inguinal	MSM	None	None	Syphilis (Treated)	None
Case 6^6^	37	M	Negative	Rash, fever, dyspnoea, and decreased exercise tolerance 13 days after a sexual encounter	Yes	NR	Yes	NR	B/L inguinal	Unclear but with multiple Partners	Yes	None	Syphilis (Treated)	None
Case 7^5^	21	M	Negative	Fever with anal pain four days after unprotected homosexual intercourse	Yes	NR	NR	NR	None	MSM, Several sexual intercourses with at risk partners during the previous month	None	None	None	None
Case 8^5^	25	M	NR	Pustules on face and penis a few days after unprotected sexual intercourse	NR	NR	NR	NR	None	Unclear	None	None	None	None
Case 9^5^	32	M	NR	Erosive cutaneous lesions on the penis one week after unprotected sexual intercourse	Yes	NR	NR	NR	None	Heterosexual	None	None	None	None
Case 10^11^	40	M	NR	Odynophagia, a swollen right submandibular lymph node, cervical pain, and a fever of up to 38◦C.	Yes	NR	NR	NR	Rt. Sub-mandibular	MSM, mainly oral. Partner diagnosed with genital Mpox	None	None	HPV Condyloma	None
Case 11^10^	45	M	Negative	Recent onset of pustular lesions	NR	Yes	Yes	NR	Painful U/L inguinal	MSM	No, but history of ARVT use	None	None	HIV
Case 12^7^	Middle aged	M	NR	Sore throat, fever, myalgias, headache, chest pain.	Yes	Yes	NR	Yes	B/L Painful inguinal	MSM	No, but history of ARVT use	Asthma, OSA	None	HSV-2
Case 13^7^	Young adult	M	NR	Flu-like symptoms, constant, non-radiating, dull, left-sided groin pain and rectal pain	Yes	Yes	NR	Yes	Rt. tender inguinal	MSM	None	OSA, external hemorrhoids, Obesity	Syphilis	HSV-2

**Table 3:** Summary of cardiac manifestations and relevant investigations carried out in 13 patients discussed in our review

Cases	Nature of Chest Pain	ECG Findings	TTE	VEF	Echo Findings	CMR Findings	Blood tests	Chest X Ray	Diagnosis
Case 1^8^	Constant, sharp, nonradiating, pleuritic chest pain, relieved when sitting upright and worse when lying down	Concave ST-elevations	NR	44%	↓LV EF	Myocardial edema	↑TLC, ↑Neutrophils ↑Monocytes ↑CRP ↑Troponin	Non-specific retro-cardiac opacities.	Acute myocarditis
Case 2^9^	Chest tightness radiating to the left upper extremity	Repolarization abnormalities	Preserved biventricular systolic function and no pericardial effusion	56%	U/R	Myocardial edema and enhancements likely due to necrosis	↑CRP ↑CPK ↑Troponin ↑BNP	Normal cardiothoracic index, and no interstitial infiltrates, pleural effusion, or masses	Acute myocarditis
Case 3^12^	Retrosternal chest pain radiating to the left arm. Chest tightness after the commencement of the pain	Widespread ST-elevations	Good bi-ventricular function with preserved systolic ejection fraction over 55%, hyperdynamic systolic function	NR	Mild pericardial effusion	NR	↑CRP ↑ESR ↑TLC	No acute pathology	Pericarditis
Case 4^13^	Central, nonradiating, pressure-like chest pain	ST changes consistent with myopericarditis	NR	61%	LV dysfunction	Mild pericardial effusion Focal edema	↑Troponin ↑CPK	NR	Myopericarditis
Case 5^6^	Chest pain and dyspnoea	Normal	NR	69%	U/R	NR	↑Troponin ↑ Pro-NT-BNP	U/R	Acute myocarditis
Case 6^6^	Dyspnoea and decreased exercise tolerance without chest pain	T wave inversions in the inferior and anterolateral leads	NR	NR	U/R	NR	↑Troponin Normal BNP	NR	Acute myocarditis
Case 7^5^	Acute chest pain radiating to the arms and jaw	ST elevation in inferior leads	Nondilated, nonhypertrophied left ventricle with segmental hypokinesis of inferior and inferolateral walls	56%	Segmental hypokinesis	No cardiac inflammation at four weeks	↑Troponin ↑CPK ↑CRP	NR	Acute myocarditis
Case 8^5^	Constant chest pain and palpitations	ST elevation in the inferior and anterior territories	inferolateral akinesia	45%	↓LVEF	NR	↑Troponin ↑CRP	NR	Acute myocarditis
Case 9^5^	Retrosternal chest pain and fever	Normal	Normal	NR	Normal	Segmental myocarditis on 11th day	↑Troponin ↑CPK ↑CRP	NR	Acute segmental myocarditis
Case 10^11^	Oppressive epigastric pain extending to the chest	Concave ST-elevations, negative T wave, and depressed PR segment	Normal	Normal	NR	Myocardial edema Subepicardial and mesocardial enhancements and mild signs of pericarditis	↑CRP ↑Troponin	No significant findings	Acute myopericarditis
Case 11^10^	NR	NR	NR	NR	NR	NR	↑CPK ↑Lymphocytes ↑AST ↑Lactate dehydrogenase	NR	Uncertain
Case 12^7^	Intermittent pleuritic chest pain	Normal	NR	NR	NR	NR	↑Troponin	NR	Demand ischemia
Case 13^7^	Sharp chest pain that resolved spontaneously	Normal	NR	NR	NR	Normal	↑Troponin	NR	Demand ischemia

## 7. Patient Characteristics

Age of the patient was reported in 10/13 cases with a median age of 33 years ranging from 21 to 51 years. All of the patients included in this review were men. Out of the 13 patients, three were reported to be positive for the Human Immunodeficiency Virus (HIV)^7,10,13^, while three had been previously treated for Syphilis^6,7^, and one had previous history of Human Papillomavirus Condyloma^11^. Pre-exposure prophylaxis for HIV was reported in two patients^6,9^. Moreover, asthma and obstructive sleep apnea (OSA) were reported comorbidities in one patient^7^, and one case had been previously treated for OSA, external hemorrhoids, and obesity^7^.

## 8. History of Sexual Contact

A total of 53.84% (7/13) patients had a history of sexual contact with a same sex partner^5-7,10,11,13^. Two patients reported a history of sexual contact with a partner of opposite sex^5,8^. One patient reported having sexual contact with multiple partners^6^. Sexual history in the remaining patients was unclear.

## 9. Clinical Presentation

Patients most commonly presented with fever^5-9,11-13^, myalgia^7-10,13^, malaise^6,9,10,12^, and headache^6,7,13^. Classical cutaneous lesions which were umblicated^6-8,11,13^, erythematous^7^, pustular^5,6,9^, vesiculo-pustular^6,12,13^, vesicular^6,10^, papular^13^, and erosive^5^, in nature were reported in almost all cases with wide ranging distribution patterns as shown in Table 4. Lymphadenopathy was reported in 69.23% (9/13) of the cases with inguinal being the most common site^6-10^. Submandibular^11,13^, and cervical lymphadeno-pathy^6^, was also reported. Out of these nine patients with lymphadenopathy, 55.55% (5/9) were reported as having painful or tender lymph node enlargement^7,8,10,13^.

**Table 4:** Summary of novel presentations and cutaneous lesions described in 13 patients included in our review

Cases	Novel Presentations	Cutaneous Lesions	Characteristics of cutaneous lesions	Distribution
Case 1^8^	None	Yes	Umblicated and ulcerated	Genital
Case 2^9^	None	Yes	Pustular and ulcerated	Face, limbs, and genitals
Case 3^12^	None	Yes	Vesiculopustular	Face and limbs
Case 4^13^	None	Yes	Umblicated, Papules, and vesiculopustular	Genital, trunk, and upper lip
Case 5^6^	None	Yes	Vesiculopapular, pustular, and ulcerated with erythematous borders	Disseminated and genital
Case 6^6^	None	Yes	Umblicated and vesicular	Upper limb and pubis
Case 7^5^	Anal pain	NR	NR	NR
Case 8^5^	None	Yes	Pustules	Face and genitals
Case 9^5^	None	Yes	Erosive	Genitals
Case 10^11^	Pharyngitis	Yes	Umbilicated	Trunk and proximal limbs
Case 11^10^	Sore throat	Yes	Vesicular and ulcerated	Genital and diffuse
Case 12^7^	Oropharyngeal	Yes	Erythematous rash and some umblicated	Chest, back, extremities, and genitals
Case 13^7^	Proctalgia, perianal irritation	Yes	Erythematous	NR

A total of four patients reported novel clinical presentations documented in the current Mpox outbreak. These included pharyngitis^11^, sore throat^7^, proctalgia^5,7^, and perianal irritation^7^. Some patients were also concurrently diagnosed with STDs along with Mpox, which included chlamydia and Herpes simplex virus-2 (HSV-2)^7^.

## 10. Cardiac Manifestations

Most of the patients reported chest pain as the primary manifestation of cardiac system involvement^5-8,12,13^. Other presentations included chest tightness^9^, dyspnoea^6^, palpitations^5^, and epigastric pain radiating to chest^11^. The characteristics of chest pain have been documented in Table 3 and percentage of occurrence of each symptom involving the cardiac system is given in Table 5.

**Table 5:** Percentage of occurrence of cardiac symptoms in 13 patients discussed in our review

Cardiac Manifestation	Percentage occurring
Dyspnea	15.38% (2/13)
Acute chest pain	69.2% (9/13)
Chest tightness	15.38% (2/13)
Palpitations	7.69% (1/13)
Decreased exercise tolerance without chest pain	7.69% (1/13)

## 11. Investigation Findings

Elevated troponin was the most commonly reported investigation finding, which was observed in 84.6% (11/13) of the patients^5-9,11,13^, followed by an elevated C-Reactive protein (CRP) which was reported in 53.84% (7/13) of the patients^5,8,9,11,12^. Some of the other reported investigation findings were elevated creatine phosphokinase (CPK)^5,9,10,13^, Pro-N type brain natriuretic peptide^6^, brain natriuretic peptide^9^, total leukocyte count^8,10,12^, Erythrocyte sedimentation rate^12^, aspartate transaminase^10^, and lactate dehydrogenase^10^. A total of six patients had ST-elevations on electrocardiogram (ECG)^5,8,11-13^, while T-wave inversion^6,11^, was observed on two ECGs, PR-segment depression on one^11^, repolarization abnormalities on one^9^, and no changes on ECG were noted for four patients^5-7^. Echocardiography was performed for almost all patients and was unremarkable for 7/12 patients^5-7,9,11^, while three patients had left ventricular dysfunction^5,8,13^. Segmental hypokinesia^5^, and mild pericardial effusion^12^, were also noted on echocardiography. Out of 13, six cases report findings of cardiac magnetic resonance imaging (MRI) suggesting myocardial edema^8,9,11,13^, pericardial effusion^13^, and subepicardial and mesocardial enhancements^11^.

## 12. Diagnosis

A definitive diagnosis was provided for 10 of the 13 patients, with Mpox-associated acute myocarditis being the most common cardiac manifestation which was observed in 70% of these patients^5,6,8,9^. Other diagnosis included pericarditis^12^, and acute myopericarditis^11,13^. Two patients were labelled having ‘demand ischemia’^7^, and another patient was only noted to have an increased CPK^10^- no definitive diagnosis was reached for these three patients.

## 13. Management

Most of the patients received supportive care for acute myocarditis^6,9,13^as summarized in Table 6. One patient diagnosed with Mpox associated pericarditis received therapy with 1gm Aspirin 8-hourly for 14 days^12^. Tecovirimat was the most commonly employed antiviral medication used in this patient population^5,6,8,11^. Certain other medications used were ceftriaxone for chlamydia^8^, doxycycline for syphilis of unknown latency^6^, valaciclovir for HSV-2^7^, dexketoprofen^11^, colchicine^11^, bisoprolol^5^, and ramipril/angiotensin converting enzyme-inhibitors^5,8^. Almost all of the patients recovered without complication.

**Table 6:** Summary of management of 13 cases included in our review

Cases	Other causes ruled out	Monkeypox Diagnosed	Management	Resolution of lesions/symptoms	Hospital Stay	Final Outcome
Case 1^8^	Yes	Yes	Tecovirimat Ceftriaxone and Azithromycin ACE inhibitor	Skin lesions and lymphadenopathy resolved in nine days	10 days (LAMA)	Uncertain
Case 2^9^	Yes	Yes	Supportive care	Complete	Seven days	Discharged upon resolution
Case 3^12^	Yes	Yes	Supportive care + 1gm Aspirin/8 hourly for 14 days	On seventh day	Seven days	Discharged with high dose aspirin prescription
Case 4^13^	Yes, except EBV, CMV, Parvovirus B19, Chagas	Yes	Supportive care	LV function restored on fifth day Full recovery on 25th day	NR	Full recovery
Case 5^6^	Yes	Yes	Tecovirimat Doxycycline for syphilis of unknown latency No specific treatment for myocarditis	Chest pain resolved at day two, Skin lesions resolved at day 10	10 days	Discharged with isolation precautions
Case 6^6^	Yes	Yes	Supportive care	Dyspnea resolved on fourth day	Four days	Discharged after isolation precautions
Case 7^5^	Yes	Yes	Bisoprolol and Ramipril	No recurrence of pain	NR	NR
Case 8^5^	Yes	Yes	Bisoprolol and Ramipril	Rapid and favourable	NR	NR
Case 9^5^	Yes	Yes	Bisoprolol Tecovirimat	Chest pain resolved on day two	NR	NR
Case 10^11^	Yes	Yes	Dexketoprofen Colchicine Tecovirimat	Skin lesions and lymphadenopathy resolved in nine days	NR	Discharged with instructions to follow with a CMR on 20th March, 2023
Case 11^10^	Yes	Yes	NR	NR	NR	NR
Case 12^7^	NR	Yes	Unclear Valacyclovir for HSV2	NR	NR	Discharged with instructions to quarantine
Case 13^7^	NR	Yes	Unclear Valacyclovir for HSV2	NR	NR	Discharged with instructions to quarantine

## 14. Discussion

Given the atypical clinical manifestations, diverse modes of transmission and rapid spread from endemic to non-endemic areas, Mpox is emerging as a significant global health concern^14^. Thus, it is important not to undervalue the onset of chest pain in an infected patient, which should trigger immediate cardiac investigations.

### 14.1. Cardiovascular events following vaccination

There appears to be a considerable overlap in the antigenic properties of various Orthopoxviruses such as variola virus, cowpox virus, and the Mpox. Several reports have documented that patients with hemorrhagic smallpox can develop acute onset late-stage myocarditis^15^. These findings have also been replicated in Crab-Eating Macaque model using the cowpox virus which mimics hemorrhagic smallpox in humans^15^. Furthermore, smallpox vaccine is strongly linked to post-vaccination myocarditis, with a reported incidence of 12 per 100,000 vaccinations^16^. The incidence of myocarditis following administration of ACAM2000 is 3.6 times greater among the vaccinated USA military personnel^17,18^. Within this group, definite vaccinia myocarditis was reported in 1 out of every 10,000 vaccinated individuals, typically manifesting 8 to 14 days after receiving the smallpox vaccine. In addition to these, less frequently reported adverse events involving the cardiac system include dilated cardiomyopathy and cardiac ischemia^17,18^. The vaccine against smallpox called the ‘JYNNEOS’ is prepared using the Vaccinia virus as the inoculating agent and has a reported efficacy of 85% against Mpox^8^. As a result of these viruses’ similar antigenic or immunogenic properties, it can be extrapolated that Mpox may also cause myopericarditis via direct cardiac tropism or by an immune-mediated response.

### 14.2. Cardiac Manifestations of Mpox

As indicated by the results of our scoping review, myocarditis was the most prevalent cardiac lesion among the 13 patients included in our review with acute chest pain as the most frequently reported symptom of cardiac system involvement. In addition to myocarditis and pericarditis, recently published literature reports congestive heart failure^17^, arrythmias^19^, and vascular complications^20^ following Mpox infection.

#### 14.2.1. Pathogenesis of Mpox-induce Myocarditis

The mechanism by which Mpox may cause inflammation of the myocardium remains largely unclear given the limited data available regarding Mpox associated myocarditis. The primary pathogenic process of viral myocarditis involves lymphocytic infiltration coupled with myonecrosis, which usually becomes evident at around 10 to 14 days following infection^21^. Previously published literature reports direct damage caused by viral antigens as one of the possible mechanisms of myocardial injury. The virus may directly invade the myocardium and undergo replication within the cardiac myocytes, resulting in inflammatory disease^22,23^. However, an absence of evidence of direct viral infection of myocardial cells observed on histopathological investigation suggests that the mechanism of myocarditis in Mpox and other orthopoxvirus infections may be immune-mediated^6,24^. Furthermore, the most frequently reported histologic finding associated with viral myocarditis is infiltration of the myocardium with lymphocytes. Additionally, edema of myocardial interstitium has also been reported. This is further suggestive of the immune-mediated pathophysiology of Mpox induced myocarditis^23^. An inflammatory response, characterized by the infiltration of immune cells, release of proinflammatory cytokines, and activation of cytotoxic T cells, results from the binding of viral genome to the immune cells that triggers the immune system^22^. This complex interplay between direct damage induced by the virus and the immune response is likely to contribute to the development of myocardial inflammation and subsequent impairment of cardiac function^25^. This inflammation of the myocardium may be self-limiting or progress to severe fulminant myocarditis^26^. Further research is warranted to achieve a thorough understanding of the pathological mechanisms underlying myocarditis development in individuals with Mpox.

#### 14.2.2. Pathogenesis of Mpox-induced Pericarditis

Pericarditis refers to the inflammation of the pericardium surrounding the heart. As indicated by our findings, as well as previously published literature, Mpox can cause pericarditis through direct invasion and immune mediated mechanisms^22^. The pericardium may be directly invaded by the virus or an immune response following viral invasion may be observed^27^.

The differential diagnoses of inflammatory cardiac complications in presence of mucocutaneous lesions and lymphadenopathy are vast, hence the diagnosis of these complications in Mpox cases becomes challenging. Thus, it is imperative to maintain a broad perspective when encountering such presentations. Considering the recent surge in cases and diagnostic uncertainty associated, a criterion is overdue to escalate the clinical suspicion of cardiac complications in patients with suspected or diagnosed Mpox.

### 14.3. Investigations and Management

The following clinical characteristics should prompt further investigations to identify Mpox as the underlying cause: 1) Diagnostic criteria satisfying clinically suspected myocarditis/pericarditis; 2) ECG showing ST changes, T-wave, or PR-segment abnormalities; 3) Temporally related to constitutional symptoms of a viral infection such as fever, myalgia, fatigue, headache, and/or other flu-like symptoms; 4) Painless or painful umblicated, vesiculopapular, and/or vesiculopustular lesions on skin with local lymphadenopathy; 5) Other novel manifestations of Mpox such as ocular, oropharyngeal, and/or anorectal; 6) History of sexual contact with a male or female partner within the last 3-17 days; 7) Common differentials ruled out via serology or Polymerase Chain Reaction (PCR). Investigations such as complete blood count, CRP, and cardiac troponins along with electrocardiogram and echocardiography play a crucial role in reaching the diagnosis of myopericarditis. Though Endomyocardial biopsy (EMB) is the gold standard for the diagnosis of myocarditis, it is not commonly performed given the invasive nature of the procedure^9^. Therefore, cardiac MRI remains the non-invasive gold standard for the prompt diagnosis of myocarditis^9^. Although the treatment strategy adopted will depend mainly on the severity of the presenting symptoms as well as the presence of any complications, the management of Mpox-induced cardiac lesions remains largely supportive^28^. For mild cases of viral myocarditis, supportive measures include taking rest, maintaining fluids and reducing inflammation^6^. In considerably severe cases, hospital admission with strict monitoring, and advanced interventions may become mandatory. Anti-inflammatory agents and immunosuppressive drugs may be employed^29^. For pericarditis, the use of NSAIDs, colchicine, corticosteroids in addition to supportive measures have been recommended^30^. Our results show that almost all patients made full recovery with no reported complications, but myopericarditis may have a significant impact on the prognosis as well as the quality of life of the patients, thus highlighting the need for accurate diagnosis^31^. Despite the positive outcome of myocarditis in some patients, caution is advised as the limited number of reported cases calls for further research. Thus, individuals with Mpox should promptly seek medical attention if they have chest pain. In countries with Mpox outbreaks, healthcare providers should consider Mpox as a potential cause if at-risk patients are diagnosed with myocarditis^5^.

### 14.4. Limitations

Although our review provides a broad overview, it does not present the depth of the existing literature and thus makes the conduct of a meta-analysis unlikely. However, in light of our objective this methodology seems appropriate. Currently, there is only limited literature available in the form of case reports and case series and given the continuously evolving nature of novel Mpox manifestations, it is unlikely that our results provide a comprehensive and generalizable picture of the cardiac manifestations of Mpox.

Additionally, quality assessment of the case reports meeting eligibility criteria was not performed since it falls beyond the scope of a scoping review. A descriptive summary of the results has been presented without any statistical aggregation of extracted data which is beyond the objectives of this scoping review. Overall, this scoping review provides a broad overview of the topic and calls attention towards the limitations of the existing literature.

### 14.5. Future Recommendations

Our scoping review has revealed a significant dearth of high-quality studies investigating the cardiac system involvement in the current outbreak of Mpox. The studies that were eligible for inclusion in our review were primarily limited to case reports and case series, highlighting the need for more rigorous research in this area. The current literature lacks information on potential risk factors that may predispose certain patient populations to cardiac complications associated with Mpox, as well as factors that may predict progression to severe disease. Additionally, there is a need to gain a deeper understanding of the underlying pathology of the cardio tropism of Mpox and the immunologic response generated by the virus in order to develop effective preventive and therapeutic strategies. The indications for antiviral therapy in patients with Mpox and cardiac involvement, as well as the choice of antiviral for these patients, remain unresolved questions.

## 15. Conclusion

Through this scoping review, we draw attention to the paucity of relevant data and advocate for increased research efforts to better understand the cardiac manifestations of Mpox. Larger, well-designed studies are necessary to establish a link between monkeypox and myocarditis and determine the course of disease and development of complications. It is worth noting that Mpox, like many viruses, exhibits a diverse range of manifestations, and prompt action is necessary to effectively manage and prevent inflammatory cardiac complications associated with Mpox.

## KEY POINTS

**◊** *It is crucial to recognize novel cardiac manifestations in case of the current Monkeypox outbreak, to guide comprehensive surveillance, preparedness, and response efforts.*

**◊** *This knowledge gap emphasizes the urgency for more comprehensive studies evaluating immune response, risk factors, and disease progression to guide better prevention and treatment strategies.*
